# Risk Assessment of Oxidative Stress Induced by Metal Ions Released from Fixed Orthodontic Appliances during Treatment and Indications for Supportive Antioxidant Therapy: A Narrative Review

**DOI:** 10.3390/antiox10091359

**Published:** 2021-08-26

**Authors:** Jasmina Primožič, Borut Poljšak, Polona Jamnik, Vito Kovač, Gordana Čanadi Jurešić, Stjepan Spalj

**Affiliations:** 1 Department of Orthodontics and Jaw Orthopedics, Medical Faculty, University of Ljubljana, Vrazov trg 2, SI-1000 Ljubljana, Slovenia; jasmina.primozic@mf.uni-lj.si; 2 Laboratory of Oxidative Stress Research, Faculty of Health Sciences, University of Ljubljana, Zdravstvena pot 5, SI-1000 Ljubljana, Slovenia; kovacv@zf.uni-lj.si; 3 Biotechnical Faculty, University of Ljubljana, SI-1000 Ljubljana, Slovenia; polona.jamnik@bf.uni-lj.si; 4 Department of Medical Chemistry, Biochemistry and Clinical Chemistry, Faculty of Medicine, University of Rijeka, 51000 Rijeka, Croatia; gordanacj@medri.uniri.hr; 5 Department of Orthodontics, Faculty of Dental Medicine, University of Rijeka, 51000 Rijeka, Croatia; stjepan.spalj@fdmri.uniri.hr

**Keywords:** fixed orthodontic appliances, oxidative stress, metal ions, risk assessment, antioxidant therapy

## Abstract

The treatment with fixed orthodontic appliances could have an important role in the induction of oxidative stress and associated negative consequences. Because of the simultaneous effects of corrosion, deformation, friction, and mechanical stress on fixed orthodontic appliances during treatment, degradation of orthodontic brackets and archwires occurs, causing higher concentrations of metal ions in the oral cavity. Corroded appliances cause the release of metal ions, which may lead to the increased values of reactive oxygen species (ROS) due to metal-catalyzed free radical reactions. Chromium, iron, nickel, cobalt, titanium, and molybdenum all belong to the group of transition metals that can be subjected to redox reactions to form ROS. The estimation of health risk due to the amount of heavy metals released and the level of selected parameters of oxidative stress generated for the time of treatment with fixed orthodontic appliances is presented. Approaches to avoid oxidative stress and recommendations for the preventive use of topical or systemic antioxidants during orthodontic treatment are discussed.

## 1. Transition Metal Ions, Fenton-like Chemistry and Oxidative Stress

Oxidative stress was first explicated by Sies [1] as “a disturbance in the balance between pro-oxidants and antioxidants in favor of the former, leading to potential damage”. Oxidative stress occurs in case of significant imbalance between the production and degradation of reactive oxygen species (ROS) with prominent pro-oxidants, generating possible oxidative damage. ROS comprise both free radicals and non-free radical oxygen-containing molecules, such as hydrogen peroxide (H_2_O_2_), superoxide (O_2_^−^), singlet oxygen (1/2O_2_), and the hydroxyl radical (HO^−^) [2]. The cell undergoes the oxidative stress, which is the result of the activity of the ROS generating reactions as well as the efficiency of the ROS scavenger system composed of endogenous and exogenous antioxidants. Endogenous defense system includes enzymatic and nonenzymatic antioxidants, such as superoxide dismutase (Mn-SOD, Cu/Zn-SOD, and extracellular (EC)-SOD), glutathione peroxidase, catalase, peroxiredoxins, glutathione (GSH), thioredoxin, uric acid, and a system for repairing oxidative damages of molecules [3]. On the other hand, the antioxidant defense can be raised through ingested food. Exogenous antioxidants contain vitamin E and C, polyphenols, carotenoids and minerals, such as zinc and selenium. They all play a significant role in the preservation of intracellular redox status and avoidance of oxidative stress-induced damage [4]. The elevated ROS production is due to endogenous (increased mitochondrial leakage, increased O_2_ concentration, and inflammation) and exogenous causes (pollution of the environment, physically demanding sport activities, diet, chronic inflammation, smoking, psychological and emotional stress, and other) [3,5,6]. Free metal ions are yet another source of increased oxidative stress since certain metals (e.g., chromium, iron, copper, cobalt, and vanadium) have the potential of redox cycling. During this process, the metal ion can accept or donate a single electron. This process catalyzes reactions in which reactive radicals are generated and reactive oxygen species can be formed. Reduced forms of redox-active metal ions play a part in the Fenton reaction where the hydroxyl radical (HO) is produced from hydrogen peroxide.

Metal^(n+1)^ + H_2_O_2_ → Metal^(n+1)+^ + HO^∙^ + HO^−^       (1 Fenton Reaction) (1)

Additionally, the Haber–Weiss reaction between the oxidized forms of the redox-active metal ions and the superoxide anion produces the reduced form of the metal ion, which can be coupled with Fenton chemistry to produce the hydroxyl radical.

Metal^(n+1)+^ + O_2_^∙−^ → Metal^(n+1)^ + O_2_         (2 Haber-Weiss Reaction) (2)

Both O_2_^−^ and H_2_O_2_ by themselves are not overly reactive and are consequently not particularly harmful in physiological concentrations. Nonetheless, their reactions with poorly bound iron species can cause the formation of hydroxyl radicals that are exceptionally harmful and are the prime source of cellular oxidative damage [7]. In the net reaction, a molecule of hydrogen peroxide undergoes a conversion to a hydroxyl radical and water in the presence of metal ion as a catalyst. Since the free metal concentration in biological systems is frequently very low, the crucial component for the activity of Fenton chemistry in biological systems is a functional metal redox cycle mechanism [2]. It can be concluded that any increase in the content of O_2_^−^, H_2_O_2_, or the redox active metal ions will most probably lead to the elevation levels of HO by the abovementioned chemical mechanisms.

Metal ions (e.g., Fe, Cr, Ni, Co, Ti, and Mo ions), released from corroded orthodontic appliances, all fall into the category of transition metals and could be subjected to redox cycling reactions as well as cause oxidative stress [8] in their vicinity. Metal ions are released from various types of alloys present in different components, including wires, brackets, or molar bands of fixed orthodontic appliances. Indeed, nickel, iron, and chromium can each intensify free radical production through a Fenton-like reaction [9,10,11], while cadmium, mercury, nickel, and lead can trigger ROS formation via indirect mechanism [12]. Although orthodontic alloys form an oxide layer that makes them corrosion resistant, these biocompatible metal materials tend to corrode locally in the harsh, ever-changing oral environment and degrade over time, releasing metal ions into the oral cavity [13,14], contributing to the general toxicity of fixed orthodontic appliances [15]. Moreover, due to frequent mechanical loading of the appliances in the oral cavity, the protective oxide layer is being constantly disrupted, accelerating wear as well as electrochemical corrosion. In further discussions, we will attempt to answer the specific research question: do fixed orthodontic appliances pose a health risk to patients due to corrosion and biodegradation?

## 2. Fixed Orthodontic Appliances

Fixed orthodontic appliances consist of archwires, brackets, and ligatures. During treatment, the archwires are ligated into the slot of the bracket [16]. The most commonly used materials from which parts of fixed appliances are manufactured are alloys of stainless steel (SS) and nickel-nitanium (NiTi) because of their advantageous mechanical properties. Less often, archwires made of other materials (e.g., cobalt-chromium alloy, ß-titanium (ß-Ti) alloy) are used. Orthodontic fixed appliances are usually in the oral cavity for extended time periods and are, therefore, exposed to degradation processes. The degradation process of dental metal alloys is the subject of several in vitro researches in which different parameters and their possible synergistic effects are analyzed, although a complete mapping of the oral cavity is hardly achieved due to the complex intraoral processes [17]. As a result of the ongoing temperature and pH changes, nutrition, breakdown of food and cells, varying oral flora, and the presence of plaque, various products are processed that can destroy the surface of fixed orthodontic appliances [18,19,20,21]. Metal alloys in the oral cavity are simultaneously subjected to corrosion and mechanical stress, e.g., due to masticatory forces, and in orthodontic treatments also due to the sliding of the archwire along the bracket slot. Therefore, the contact of the bracket, archwire, and ligature in the electrolytic fluid (saliva) leads to a faster and more intensive corrosion process on the metal surface [22]. Simultaneous exposure of fixed orthodontic appliances to corrosion, deformation, friction, and mechanical stress during treatment results in degradation of orthodontic brackets and archwires, which can result in elevated concentrations of metal ions in the oral cavity [23,24]. Mucosal erythema, allergic contact dermatitis, contact stomatitis, periodontitis, gingival hyperplasia, glossitis, gingivitis, and multiform erythema were all noticed during orthodontic treatment, which could be generated also by the toxic action of metal ions relieved by fixed orthodontic appliances [25,26]. As reported by the American Board of Orthodontics, treatment with fixed appliances continues for about 24 months [27]. Therefore, the safety of such appliances should be well studied as metal ions can induce either minor or major toxic effects locally (e.g., on oral cavity tissues) [28] or even systemic effects when they are absorbed and enter the systemic bloodstream [29,30].

## 3. Metal Ions Release during Treatment with Fixed Orthodontic Appliances

Nearly all fixed orthodontic appliances are constructed of metal alloys, which over certain period of time release these metal ions into the environment [31]. Among metal alloys, stainless steel, cobalt-chromium, NiTi, and β-titanium alloys are the most frequently used orthodontic materials [32]. The type of alloy, the exposure time and the environment are factors that impact the amount of metal ions released. Electrochemical corrosion, oxidation, and friction caused by continual sliding of the wires in the bracket slots impact the degradation of surface (tribocorrosion) and, subsequently, elevate the amount of ions that are released into saliva from the fixed appliances [33].

The study by Kovac et al. analyzed the amount of the metal ions released from various segments of orthodontic appliances (brackets, wires, and molar bands) composed of diverse alloys (SS, NiTi, β-Ti, and Co-Cr-Ni) over a three-month incubation period in an artificial oral environment. The results of the study show that the amounts of metal ions released from orthodontic appliances in an artificial oral environment were at a lower level than the upper intake limits. Not even one of the studied released metal ions exceeded the recommended daily dose concentrations. Molar bands and stainless-steel brackets discharged quadruple the number of ions in comparison to the stainless-steel wires. It was also observed that Ni-Ti wire released larger amount of Ni than the wires manufactured from stainless steel. Additionally, Ti-Mo wires released the lowest amount of metal ions. In terms of kinetics, metal release rates over 90 days were nonlinear, with significantly higher release in the first seven days of exposure as compared to the final seven-day exposure period.

Mikulewicz et al. [34] reported that the concentration of metal ions in artificial saliva reached 573 for Ni, 101 for Cr, 68 for Mn, and 2382 for Fe (in micrograms per liter). The increased amounts of specific metals could be attributed to the fact that Mikulewicz et al. study design constituted continuous stirring of the samples at 120 rpm during a one-month period while the samples in the study by Kovac et al. (2021) were only slightly shaken before sampling.

The release of Ni ions from different types of archwires used in orthodontics containing NiTi, SS, Cu-NiTi, and ion-implanted NiTi was studied by Charles et al. [35]. The amount of Ni ions released in all test solutions did not exceed the critical level that may trigger allergy and is found to be lower than the amount in the average daily intake. The study by Hussain et al. [36] compared nickel release from SS and NiTi archwires in artificial saliva over the period of three months using simulated fixed orthodontic appliances. The nickel release from NiTi archwires was 4.85 ng/mL and was higher in comparison to SS archwires, in which 0.41 ng/mL Ni ion release was detected. Hwang et al. [37] and Kutha et al. [38] corroborated the findings of Mikulewicz et al. for Ni release, while Barrett et al. [39] and Park et al. [40] observed significantly increased Ni levels. On the contrary, Gürsoy et al. [41] identified a lower concentration of Ni in the range of 20 μg/L.

Ortiz et al. [25] and Kuhta et al. [38] compared a NiTi archwire with an SS archwire and reported similar results on Ti release as reported by Kovac et al. Both studies found that Ti-containing alloys were more biocompatible than others. Kuhta et al. found that the difference in pH had a significant impact on ion release and that ion release was conditioned by archwire composition. It is, however, not in proportion to the metal composition of the archwire. Hwang et al. [37] determined metal release from fixed orthodontic appliances soaked in artificial saliva and noted that the values of Fe and Cr ions released from SS orthodontic appliances were even above the values reported by Kovac et al. Besides, a decrease in metal release with increasing immersion time was noted after a three-month period. Similarly, Kuhta et al. measured the highest amount of ions released in the first seven-day immersion of appliance. Hussain et al. [42] observed that ion release depends more on the type of alloy and the process of its manufacture than on the amount of ions in the alloy. According to He et al., saliva proteins influence the alloys metal ion release and have also an important role in corrosion resistance improvement [43].

There is a strong evidence that the results from in vitro studies investigating the release of metal ions over 30 days vary widely, e.g., some orthodontic alloys could not be ascertained or were as high as 7000 ng/mL [44]. The observed differences in the results regarding the quantity of metal ions released are due to different study designs, material analyzed, electroplated coatings, and different compositions of the alloys, the immersion media and different techniques and methods used at various times. On the other hand, metal ion concentrations from in vivo experiments were much lower and with less pronounced release variability, in the range between 4 and 30 ng/mL [45]. However, it should be emphasized that the metal ion concentration closer to the orthodontic alloy may be much higher than that measured in saliva, due to the constant saliva flow.

## 4. Cytotoxicity

Various methodological approaches have been used to treat cells with heavy metals. Namely, immersing orthodontic components in medium allowing immediate contact with the cells or incubating the orthodontic components in medium (e.g., artificial saliva) and dissolving out metal ions have been used to treat the cells. Another approach was reported using ingredients, such as metal chlorides, in which all metal ions acting on an orthodontic alloy are released identically, allowing the correct metal ion concentrations to be selected.

Additionally, in vitro toxicity of archwires have been performed on different cell cultures including fibroblasts [46,47,48,49], osteoblasts [46,47], smooth muscle cells [50,51], and neurons [52]. Results on SS, Ni, and Cr treated cells were generally toxic [48,52], while the results on NiTi archwires were inconsistent, with indications that it is both toxic [50,51] and non-toxic [46,47]. In the study of Spalj et al. [53], NiTi orthodontic archwire induced the lowest viability of mouse fibroblast cells, while SS archwires revealed the highest viability. The lowest inhibition of cell growth was observed in SS and TiMo, lower than in NiTi, CoCr, and the positive control.

The toxicity of metal ions employed in dental alloys was evaluated in the Saccharomyces cerevisaie yeast cultivated in various media. Metal toxicity was lower in the nutrient rich media (containing yeast extract and peptone) compared to synthetic complete media. In synthetic media, toxicity was the highest for HG, followed by Ag and Au, while Cu, Ni, Co, and Zn exhibited the lowest toxicity. The authors attributed this reduced toxicity to the sequestration of metal ions by various components in the yeast growth media [54].

Metal mixtures, simulating different orthodontic alloys and prepared in four different concentrations, were employed in the treatment of yeast cells for the period of 24 h in the study of Kovac et al. [32]. At 1000 µM concentrations, each simulated orthodontic alloy was cytotoxic, and in the instance of the CoCr alloy, even the 10-fold lower concentration was cytotoxic to S. cerevisiae cells. Mutant yeast cells showed a negative effect at the 100 µM concentration of all metal ion combinations, with the exception of SS. Cytotoxicity of orthodontic materials (brackets, archwires, resin, elastomers, and silver solder) using multiple yeast mutants either with antioxidant defense or DNA repair deficiency as a model organism was assessed also by Limberger et al. [55]. The results revealed significant cytotoxicity of silver solder, possibly induced by oxidative stress.

On the contrary, no major distinction in cell viability was identified among ceramic, metal, and polymeric conventional brackets on murine fibroblast cells L929 in the period following the in vitro exposure to four types of self-ligating and three types of conventional brackets [56]. Fixed orthodontic appliances (a combination of brackets and archwires) were examined in buccal mucosal cells. Decreased cellular viability, induced DNA damage, and elevated Ni and Cr levels were observed three months after the treatment [57]. Cicedo et al. [58] studied how metals (iron, chromium, aluminum, cobalt, copper, molybdenum, nickel, vanadium, niobium, and zirconium) released from implants effect human CD4+ T lymphocytes. All the examined metals caused T cell apoptosis at doses that were below the level causing the DNA damage. Ni induced apoptosis at 100 µM, while Co inhibited viability at a concentration of 500 and proliferation at 100 µM. An in vitro evaluation of the cytotoxicity and genotoxicity of a commercial Ti alloy for dental implantology was performed by Velasco-Ortega et al. [59]. The results showed that the Ti alloy (Ti-6Al-4V) was not cytotoxic or genotoxic in none of the conducted tests. Comparable data were obtained by Kovac et al. [32] on Ti-containing Ni-Ti and β-Ti ion mixtures, which showed no evidence of toxicity and oxidative stress formation.

NiTi shape memory alloys used in orthodontics were assessed by electrochemical assays in artificial saliva and in vitro biological tests with L132 cells and HEPM cells. The results proved the corrosiveness and cytotoxicity of Ni, as 425 µM of pure Ni ions resulted in 50% viability in human epithelial embryotic cell lines (L132). On the contrary, Ti ions and the commixture of both did not affect cell lines up to 3750 µM concentrations [60]. Issa et al. [61] measured the citotoxicity induced by changing concentrations of several metal salts on human oligodendrocyte (MO3.13) and human gingival fibroblasts (HGF). The greatest impact on survivability (50%) was observed in Co concentrations, succeeded by the Ni and Cr concentrations. Cd revealed the most noticeable cytotoxic effects on MO3.13 cells while Hg demonstrated the most significant cytotoxic effect on HGF in comparison to other tested metals.

## 5. Oxidative Stress and Oxidative Damage during the Treatment with Fixed Orthodontic Appliances

There are not many studies that investigated oxidative stress in the treatment with fixed orthodontic devices. Until recently, only one study determined the oxidative stress induced by orthodontic treatment at the systemic level [30], while the oxidative stress parameters were evaluated during orthodontic treatment either in saliva [62,63,64,65] or the gingival crevicular fluid [64]. Systemic oxidative stress parameters in the study of Kovac et al. [30] were evaluated in patients in the first seven-day period of treatment with fixed orthodontic appliances. The reactive oxygen species formation (ROS) as well as the antioxidant defense potential (AD) were assessed in the capillary blood samples from fifty-four male patients with malocclusion undergoing orthodontic treatment and untreated control subjects. Twenty-four hours after orthodontic treatment with fixed appliances, the ROS level was markedly elevated in the treatment group in comparison to the control group. Moreover, 24 h after archwire insertion, a significantly higher ROS/AD ratio was identified in the treatment group than in the control group. The authors concluded that the treatment with fixed orthodontic devices could cause systemic oxidative stress in the short run as the values of ROS and ROS/AD normalize during the period of one week following archwire insertion. Indeed, as revealed by Buczko et al. [62], the treatment with fixed orthodontic appliances alters the oxidant-antioxidant balance in saliva of individuals who are otherwise clinically healthy. Highest oxidative stress parameters (tiobarbituric acid reactive substance and total oxidant status) were detected in saliva within a week following the insertion of orthodontic appliance. Decrease in superoxide dismutase (SOD1) and catalase (CAT) was observed in stimulated saliva one week after treatment, while peroxidase (Px) was increased. It was noted that the total antioxidant status (TAS) was decreased twenty-four weeks after orthodontic treatment. The oxidative status index (OSI) increased a week after the treatment. The levels of selected markers of oxidative stress (malondialdehyde, ceruloplasmin, hydrogen donors) in the saliva of the treated patients prior to and after the beginning of treatment were additionally studied by Olteanu et al. [63]. Additionally, the current research shows increased markers of oxidative stress in the 24 h period, while seven days after appliance use the concentrations were close to baseline values. In contrast, Atuğ Oezcan et al. [64] observed no increase in oxidative stress markers and damage in saliva and gingival-crevicular fluid samples of patients with fixed orthodontic appliances when comparing the pretreatment results and the results at the sixth month of orthodontic treatment. Similarly, orthodontic treatment with self-ligating metal brackets did not statistically significantly affect salivary antioxidant parameters in the period of the first ten weeks of treatment [65].

During the treatment with fixed orthodontic appliances, metal ions are released, which could potentially induce oxidative stress and have a local oxidative effect, as observed in in vitro studies. Systemically, increased oxidative stress was observed only in the first seven-day period after the implementation of fixed orthodontic appliances, most likely due to the activation of endogenous adaptive stress responses and the induction of antioxidant endogenous defenses. Numerous in vitro studies have shown that orthodontic brackets as well as archwires cause oxidative stress linked to the release of heavy metals. All types of orthodontic brackets tested, disregarding the material components, are the origin of oxidative stress in vitro, as indicated by an increased concentration of the oxidative stress marker 8-hydroxy-2′-deoxyguanosine (8-OHdG) in the DNA of murine fibroblast cells L929. The highest stress levels were observed in the all-metal and polyurethane brackets [56]. In addition, all orthodontic archwires tested induced oxidative stress in vitro by measuring (8-OHdG) in DNA of murine fibroblast cells L929. Standard NiTi archwires produced the highest oxidative stress, while TiMo and SS triggered the lowest stress [53].

Different mixtures of Cr, Fe, Co, Ni, Ti, and Mo metal ions simulating either NiTi, SS, β-Ti, or CoCr orthodontic alloys were used in testing the capacity of metal-ions as oxidative stress-inducing agents using wild-type yeast Saccharomyces cerevisiae and two mutant ΔSod1 and ΔCtt1 as model organisms, to determine whether the lack of defense system from superoxide anions and H_2_O_2_ contributes to metal ion-induced toxicity [32]. Indeed, mutants lacking the Sod1-Ctt1 gene should be more sensitive to transition metal ion-induced ROS cytosolic SOD (Sod1) scavenges O_2_^−^and converts it to H_2_O_2_, which is further degraded to H_2_O and O_2_ by cytosolic catalase (Ctt1) [66]. A 1000 µM metal ion treatment with SS, Co-Cr-Ni resulted in significantly higher ROS values in all yeast strains than in the untreated control group, but the treatment using equal and lower concentrations of Ti-Mo did not significantly affect the ROS value. While SS, Co-Cr-Ni showed a significant increase in oxidative lipid damage in all yeast strains at the concentration of 1000 µM in comparison to the control group, and in the mutants (ΔSod1 and ΔCtt1) lipid oxidation presented already at 100 µM. Yeast mutants without the Sod1 gene have greater intracellular ROS levels than the Ctt1-lacking mutants although the same amount of lipid oxidation was observed in both mutants. Lipid oxidation and intracellular formation of ROS was almost twice as high in the mutants compared to the wild type.

Stainless steel bands, with (SSB) or without (NSB) silver soldered joints, were investigated for the cytostatic, cytotoxic, genotoxic, and DNA damage-inducing effects on the HepG2 and HOK cell lines [67]. After performing MTT reduction assay, alkaline and modified comet assay, cytokinesis block micronucleus assay, cytostasis assay and cytotoxicity assay, the results showed higher cytotoxicity and genotoxicity of SSB eluates in comparison to NSB samples. Ni and Fe were found in the SSB as well as NSB medium samples, while Cd, Cr, Ag, Cu, and Zn were found only in the SSB medium samples.

It can be concluded that oxidative stress during orthodontic treatment can be caused by exposure to heavy metals (e.g., Cr, Fe, Co, Ni, and Ti), both locally and systemically. It can be induced also by many other factors, including aseptic inflammation in the periodontal ligament by cause of mechanical force and inflammation of periodontal tissues as a result of improper oral hygiene [30]. We would also like to draw attention to the occurrence of a synergistic effect when cells are simultaneously exposed to two or more metal ions. This is because their toxic effect not only adds up with simultaneous exposure, but it can even be multiplied or potentiated. According to Rinčić Mlinarić et al. (2019), the collective concentration of Ni and Ti in concentrations of at least 162 μg/L is necessary for synergism, provoking moderate to strong cytotoxicity and a notable induction of free radicals [68]. Terpilovska and Siwicki reported that Cr^3+^ and Fe^3+^ show synergistic effects in cytotoxicity, genotoxicity, and mutagenicity assays, but in some other combinations metal ions can act antagonistically—Cr^3+^ and Mo^3+^ or Cr^3+^ and Ni^2+^ show antagonistic effect—Cr^3+^ protects from Ni^2+^ or Mo^3+^ toxicity [69].

## 6. Risk Assessment

The Acceptable Daily Intake (ADI), also known as the Tolerable Daily Intake (TDI), is a “measure of the amount of a particular substance in food or drinking water that can be ingested daily (orally) over a lifetime without posing a significant health risk” [70]. The ADI is based on the NOAEL (no-observed-adverse-effect level) calculated from animal studies and observations in humans. NOAEL is a dose defined on experiments and observations where no statistically or biologically significant adverse effects have been found. When a NOAEL cannot be determined experimentally, the term “lowest-observed-adverse-effect level (LOAEL)” is used [71]. The NOAEL is numerically divided by a safety factor (SF) to explicate and justify differences found in experimental animals and humans (factor of 10) and potential dissimilarities in sensitivity among humans (factor of 10). An additional factor of 10 may be considered if the NOAEL has not been established because adverse effects are detected at all dose levels tested. Instead of the NOAEL, the lowest observed adverse effect level (LOAEL) is employed to determine the ADI divided by 10-fold safety factor. The safety factor of 10 shall also apply in cases where no satisfactory results of chronic toxicological tests are available, and the NOAEL is consequently determined on the basis of subchronic studies [71]. The level of the safety factor used shall depend on the reliability of the data collected and the severity of the adverse reactions observed.

ADI (human dose) = NOAEL (experimental dose)/SF(3)

NOAEL and LOAEL values derived from toxicological studies on metal ions are shown in Table 1. It should be noted that Table 1 does not include studies investigating the leaching of metal ions from orthodontic appliances, but all kind of studies investigating the toxicity of metal ions.

**Table 1:** NOAEL *, LOAEL * values, tested concentration range, type of study (chronic vs. acute) and model organism tested to determine the toxicity of specific metal ions.

**Source of** **Metal Ions**	**NOAEL**	**LOAEL**	**Concentration (Dose)**	**Study Type**	**Model Organism**	**Ref.**
**Fe**						
FeSO_4_	10 μM	250 μM	10–1000 μM	acute toxicity	human leukocytes	[72]
	50 μM	100 μM	0, 10, 25, 50, 100 μM	acute toxicity	microglia cells	[73]
FeCl_3_·6H_2_O	3.2 μM	11.1 μM	3.2, 11.1, 111, 1111 μM	chronic toxicity	rats	[74] *
	100 µM	200 µM	0–1400 μM	acute toxicity	BALB/3T3, HepG2 cells	[75]
iron sucrose injection	1790 µM	3580 µM	360, 890, 1790, 3580, 8950 µM	acute toxicity	hemodialysis patients	[76] *
**Ni**						
NiCl_2_	27.2 mg/kg	54.4 mg/kg	3.4, 6.8, 13.6, 27.2, 54.4, 108.8 mg/kg	acute toxicity	mice	[77]
	100 µM	250 µM	0, 100, 250, 500, 600, 750, 1000 µM	acute toxicity	human HeLa cells	[78]
	10 µM	100 µM	0–10,000 µM	acute toxicity	human keratinocytes	[79]
NiCl_2_·6H_2_O	100 µM	200 µM	0–1400 μM	acute toxicity	BALB/3T3 cells	[75]
NiSO_4_	1.25 mg/kg/day	2.5 mg/kg/day	1.25, 2.5, 5 mg/kg/day	sub-acute toxicity	rats	[80]
Ni(NO_3_)_2_	150 μM	300 μM	0, 30, 75, 150, 300, 600 μM	acute toxicity	chromatin from rat liver cells	[81]
**Cr**						
K_2_Cr_2_O_7_	1 µM	10 µM	0–1000 µM	acute toxicity	human keratinocytes	[79]
	50 µM	400 µM	50, 100, 200, 400, 600, 1000 μM	acute toxicity	lymphocytes	[82]
CrCl_3_	400 µM	600 µM	50, 100, 200, 400, 600, 1000 μM	acute toxicity	lymphocytes	[82]
CrCl_3_·6H_2_O	100 µM	200 µM	0–1400 μM	acute toxicity	BALB/3T3 cells	[75]
**Mo**						
MoO_3_	100 µM	200 µM	0–1400 μM	acute toxicity	BALB/3T3, HepG2 cells	[75]
Na_2_MoO_4_	60.7 µM	121.4 µM	0, 60.7, 121.4, 242.7, 485.4, 970.9 µM	Sub- acute toxicity	mice	[83] *
MoCl_5_	100 µM	500 µM	50, 100, 500, 1000, 5000 µM	acute toxicity	human CD4þ T lymphocytes	[58] *
**Ti**						
Ti particles	13 µM	26 µM	13, 26, 52, 104, 209 µM	acute toxicity	Osteoblasts MC3T3-E1	[84] *
**Co**						
CoCl_2_	50 µM	100 µM	50, 100, 500, 1000, 5000 µM	acute toxicity	human CD4þ T lymphocytes	[58] *
CoCl_2_	/	1 µM	1–100 μM	acute toxicity	Balb/3T3 mouse fibroblasts	[85]

* Abbreviations: NOAEL no-observed-adverse-effect level; LOAEL lowest-observed-adverse-effect level.

Similarly, the U.S. EPA has introduced the Reference Dose (RfD), which by definition is the maximum acceptable oral dose of a toxic substance derived from the formula: RfD = NOAEL/UF (uncertainty factor) [86]. A useful additional measure in risk assessment is also the exposure margin (MOE), which presents the amount by which the NOAEL of the critical toxic effect exceeds the estimated exposure dose (EED), both expressed in the same units: MOE = NOAEL (experimental dose)/EED (human dose) [86]. According to Food and Nutrition Board from the Institute of Medicine, National Academy of Sciences defines Dietary Reference Intakes (DRI) as the general term for a set of reference values used to plan and evaluate the nutrient intake of healthy people. These values, which vary by age and gender, additionally include [87,88].

Recommended Dietary Allowance (RDA): “average daily intake sufficient to meet the nutrient requirements of almost all (97–98%) healthy people”.

Adequate Intake (AI): “a value based on observed or experimentally determined approximations of the nutrient intake of a group (or groups) of healthy people-used when an RDA cannot be determined”.

Tolerable Upper Intake Level (UL): “the highest daily nutrient intake that is unlikely to pose a risk of adverse health effects for almost all individuals in the general population. As intake increases above the UL, the risk of adverse effects increases”.

The derived no objection level (DNEL) is the level of exposure to a substance to which people should not be exposed [89].

Table 2 shows the limits of intake for each element, expressed in units of mass per day. Table 3 includes orthodontic studies showing the leaching of metal ions mainly in artificial saliva. The results are presented in different units (ppm, ng/mL, g/L, ng/cm^2^) and the observation period included different time intervals (1 day–90 days). The studies also differed in the fact that some examined leaching from whole orthodontic appliances, while others examined only individual parts (archwire/bands/brackets). These differences restrict the possibility of comparison of individual studies and extrapolate the data for human safety as well as the comparison of data with the limits presented in Table 2. In addition, it should be noted that the leaching of metals over time is not simply linear.

**Table 2:** Limit intake levels for selected metals.

Metal	Threshold		References
	Men	Women	
Iron	RDA * = 8 mg/day	RDA = 18 mg/dayRDA = 8 mg/day (postmenopause)	[90]
	UL * = 45 mg/day	UL = 45 mg/day	[90]
	RDA = 17 mg/day	RDA = 17 mg/day	[91]
	AI * = 11 mg/day	AI = 16 mg/dayAI = 11 mg/day (postmenopause)	[92]
	RDA = 10 mg/day	RDA = 10–15 mg/day	[93]
	DNEL * = 710 µg/kg bw/day (chronic exposure)	DNEL * = 710 µg/kg bw/day (chronic exposure)	[94]
Nickel	UL = 1.0 mg/day	UL = 1.0 mg/day	[95]
	GL = 0.26 mg/day	GL = 0.26 mg/day	[91]
	DNEL = 11 µg/kg bw/day (chronic exposure)	DNEL = 11 µg/kg bw/day (chronic exposure)	[96]
Chromium	AI = 35 μg/day	AI = 25 μg/day	[97]
	RDA = 30–100 µg/day	RDA = 30–100 µg/day	[93]
	GL = 10 mg/day	GL = 10 mg/day	[91]
Titanium	DNEL = 350 mg/kg bw/day (chronic exposure)	DNEL = 350 mg/kg bw/day (chronic exposure)	[98]
Molybdenum	AI = 45 μg/day	AI = 45 μg/day	[99]
	RDA = 65 μg/day	RDA = 65 μg/day	[100]
	RDA = 50–100 μg/day	RDA = 50–100 μg/day	[93]
	DNEL = 3.4 mg/kg bw/day	DNEL = 3.4 mg/kg bw/day	[101]
Cobalt	GL = 1.4 mg/day	GL = 1.4 mg/day	[91]
	DNEL = 29.8 µg/kg bw/day (chronic exposure)	DNEL = 29.8 µg/kg bw/day (chronic exposure)	[102]

* Abbreviations: RDA Recommended Dietary Allowance; UL Tolerable Upper Intake Level; AI Adequate Intake; DNEL The derived no objection level; ADI Acceptable Daily Intake.

The highest quantities of released metal ions were observed in the study of Mikulewicz et al. Metal concentrations observed in saliva compared to the maximum acceptable concentrations of metal ions in drinking water (80/778EEC/; 98/83/ES; 2020/2184) [103,104] should be of concern. For example, the set levels for Ni (573 μg/L) and Cr (101 μg/L) were exceeded 11 times and twice, respectively. Additionally, the concentration of Mn was (68 μg/L) 1.4 times higher and Fe (2382 μg/L) concentration was 12 times higher than the maximum acceptable values for drinking water as set in the EU Drinking Water Directive 98/83/EC. Following American standards, the concentration of Ni ions was 5.5 times higher, while other elements were at an acceptable level [105]. The volume of artificial saliva (in milliliters) that the recommended daily doses of these elements would provide, would amount to 61 for Ni, suggesting that the only possible exposure risk to orthodontic patients is from it [34], suggesting that the latter presents the sole potential exposure risk to the treated patients. Nevertheless, the amount of ions released by orthodontic appliances may induce delayed allergic reactions, which should be considered when selecting the type of archwire, with special attention to patients with metal hypersensitivity or limited oral hygiene [38]. Finally, it should be noted that the individual reaction to metal ions may occur in some individuals at much lower concentrations than indicated in the legal limits for the general population.

**Table 3:** Average concentrations of leached metal ions from orthodontic appliances.

Metal Ion Release from Orthodontic Appliances								
Fe	Ni	Cr	Ti	Mo	Co	Alloy Type	Time	Ref.
310 ± 123.1 ppb	1292 ± 437.5 ppb	0 ppb	/	/	4 ± 5.477 ppb	SS wire + SS brackets + SS band	90 days	[106]
450 ± 222.8 ppb	1864 ± 600.2 ppb	18 ± 34.9 ppb	/	/	10 ± 7.071 ppb	NiTi wire + SS brackets + SS band		
614 ± 531.7 ppb	2466 ± 867.8 ppb	30 ± 51.9 ppb	/	/	12 ± 4.47 ppb	Co-NiTi wire + SS brackets + SS band		
534 ± 558 ppb	2132 ± 1143 ppb	20 ± 44.72 ppb	/	/	12 ± 4.472 ppb	Elgiloy wire + SS brackets + SS band		
2382 ppb	573 ppb	101 ppb	/	/	/	SS wires, SS brackets, SS bands, SS metal ligatures	30 days	[34]
1107 ppb	15 ppb	8 ppb	/	/	/	control		
/	18.5 ± 13.1 ppb	2.6 ± 1.6 ppb	/	/	/	SS brackets and bands; SS + NiTi archwires	16 ± 2 months	[29]
/	11.9 ± 11.4 ppb	2.2 ± 1.8 ppb	/	/	/	control		
10.78 ± 17.66 µg	28.33 ± 29.19 µg	12.66 ± 4.61 µg	/	133.33 ± 57.7 µg	/	NiTi archwire + Dentaurum brackets	28 days	[107]
21.33 ± 8.73 µg	17.66 ± 11/59 µg	<10 ± 0 µg	/	110 ± 17.32 µg	/	NiTi archwire + American Ortho brackets		
14.33 ± 5.33 µg	76.66 ± 62.52 µg	16.66 ± 11.54 µg	/	<130 ± 0 µg	/	NiTi archwire + Shinye brackets		
<10 ± 0 µg	22 ± 7.54 µg	30 ± 0 µg	/	120 ± 17.32 µg	/	NiTi archwire + ORJ brackets		
16 ± 5.29 µg	37 ± 20.042 µg	26 ± 6.92 µg	/	110 ± 17.32 µg	/	SS archwire + Dentaurum brackets		
11 ± 1.73 µg	45 ± 31 µg	26 ± 6.92 µg	/	0 ± <100 µg	/	SS archwire + American Ortho brackets		
<10 ± 0 µg	293.2 ± 365.6 µg	15.33 ± 4.61 µg	/	0 ± <100 µg	/	SS archwire + Shinye brackets		
<10 ± 0 µg	44.66 ± 35.50 µg	15.33 ± 4.61 µg	/	110 ± 17.32 µg	/	SS archwire + ORJ brackets		
67.7 ± 9.6 ppb	10 ± 6.3 ppb	4.5 ± 0.5 ppb	2 ± 0.6 ppb	/	/	NiTi New brackets + new archwire	45 days	[41]
64.3 ± 8 ppb	15 ± 3.3 ppb	4 ± 0.7 ppb	2 ± 0.7 ppb	/	/	NiTi Recycled brackets + new archwire		
115 ± 11.9 ppb	20 ± 6.5 ppb	7.3 ± 0.6 ppb	3.5 ± 1.3 ppb	/	/	NiTi New brackets + recycled archwire		
140 ± 9.4 ppb	20 ± 3.3 ppb	7.5 ± 0,6 ppb	4 ± 0.7 ppb	/	/	NiTi Recycled brackets + recycled archwire		
/	2 ppb	/	/	/	/	NiTi archwire	28 days	[108]
/	/	/	/	/	/	TMO archwire		
289 ppb	6 ppb	11 ppb	/	/	/	SS archwire		
/	4 ppb	3 ppb	/	/	/	Cu-NiTi archwire		
/	67 ± 10.8 ppb	30.8 ± 4.3 ppb	/	/	/	SS brackets, bands NiTi & SS archwires	1.5 years	[31]
/	5.02 ± 0.001 ppb	1.27 ± 0.09 ppb	/	/	/	control		
/	0.93 ± 0.04 μg	/	/	/	/	NiTi archwires	21 days	[35]
/	0.66 ± 0.02 μg	/	/	/	/	SS archwires		
/	0.67 ± 0.02 μg	/	/	/	/	CuNiTi archwires		
/	0.77 ± 0.05 μg	/	/	/	/	Ion implanted NiTi archwires		
/	125 ± 22 μg	112 ± 18 μg	/	/	/	SS ¼ archwire + bands + brackets	12 days	[40]
96.06 ± 57.4 ppb/day	41.66 ± 33.99 ppb/day	33.43 ± 24.05 ppb/day	0 ± 0	/	/	SS archwire	1st day	[38]
25.55 ± 10.00 ppb/day	10.21 ± 2.68 ppb/day	3.83 ± 1.93 ppb/day	0 ± 0	/	/		6th day	
11.08 ± 5.89 ppb/day	5.28 ± 1.87 ppb/day	1.43 ± 0.69 ppb/day	0 ± 0	/	/		7th day	
5.62 ± 1.47 ppb/day	3.84 ± 0.86 ppb/day	0.70 ± 0.10 ppb/day	0 ± 0	/	/		14th day	
38.47 ± 15.67 ppb/day	11.77 ± 2.84 ppb/day	10.49 ± 3.90 ppb/day	0.14 ± 0.04 ppb/day	/	/	NiTi archwire	1st day	[38]
26.93 ± 5.44 ppb/day	10.83 ± 3.49 ppb/day	3.30 ± 0.95 ppb/day	0.01 ± 0 ppb/day	/	/		6th day	
13.07 ± 4.01 ppb/day	6.13 ± 1.39 ppb/day	1.76 ± 0.34 ppb/day	0.01 ± 0.01 ppb/day	/	/		7th day	
6.81 ± 1.70 ppb/day	3.38 ± 1.67 ppb/day	1.06 ± 0.21 ppb/day	0.01 ± 0 ppb/day	/	/		14th day	
21.18 ± 6,43 ppb/day	7.12 ± 1.33 ppb/day	4.39 ± 1.99 ppb/day	0.12 ± 0.02 ppb/day	/	/	Termo NiTi archwire	1st day	[38]
19.11 ± 6.28 ppb/day	7.26 ± 1.10 ppb/day	2.10 ± 0.84 ppb/day	0.01 ± 0 ppb/day	/	/		6th day	
8.79 ± 3.79 ppb/day	5.06 ± 1.57 ppb/day	1.23 ± 0.80 ppb/day	0.01 ± 0 ppb/day	/	/		7th day	
2.50 ± 0.59 ppb/day	2.33 ± 0.77 ppb/day	0.40 ± 0.15 ppb/day	0 ± 0	/	/		14th day	
42.33 ± 27.06 ppb	0.00 ± 0.00 ppb	0.36 ± 0.33 ppb	6.64 ± 2.00 ppb	0.80 ± 0.27 ppb	0.00 ± 0.00 ppb	control	30 days	[25]
520.0 ± 210.1 ppb	416.9 ± 133.5 ppb	8.97 ± 6.73 ppb	9.03 ± 2.08 ppb	3.11 ± 1.47 ppb	1.38 ± 1.25 ppb	SS brackets + SS bands		
49.67 ± 29.29 ppb	0.00 ± 0.00 ppb	0.06 ± 0.05 ppb	5.35 ± 3.96 ppb	0.11 ± 0.07 ppb	0.03 ± 0.01 ppb	Ti brackets + Ti bands		
179.49 ± 99.1 ppb	5.79 ± 2.07 ppb	0.91 ± 0.28 ppb	3.79 ± 3.11 ppb	0.60 ± 0.21 ppb	0.00 ± 0.00 ppb	Ni-free brackets + bands		

## 7. Indications for Antioxidant Therapy: Pros and Cons

The awareness that leaching of metal ions occurs in the treatment with fixed orthodontic appliances, and the evidence of the resulting elevated oxidative stress and oxidative impairment require a careful consideration of the antioxidant application in patients during treatment. Based on the present literature review and risk assessment, an increased intake of antioxidants one week before and one week (as well as after each reactivation of the device/wire change) after treatment with fixed orthodontic appliances would be advocated, while prolonged intake of synthetic antioxidants is not recommended for several reasons (reviewed in [6,109]). Although several in vitro studies indicate that supplementation with synthetic antioxidants or plant extracts reduces oxidative stress and oxidative damage (reviewed in [110]), the results of epidemiological and human studies are conflicting [111,112,113,114]. Moreover, the effect of antioxidants as free radical scavengers in vitro is well studied, but their effect on ROS -induced damage prevention in vivo is still controversial. ROS effects various vital physiological processes in a human, including pathogen defense, stress response induction, regulation of glucose, cellular growth and proliferation, and systemic signaling [115]. Both excessive “antioxidant/reductive stress” and oxidative stresses can be harmful to organisms because antioxidants have no capacity to discriminate among physiologically beneficial and harmful radicals, which induce oxidative damage to biomolecules [6]. Antioxidants can cause similar damage as oxidants when present in large concentrations or in the presence of redox cycling metal ions that can induce the creation of highly reactive free radicals through the Fenton-like reaction. For example, vitamins C and E reduce iron, which generate free radicals through the Fenton-like reaction [116,117].

2 Fe^3+^ + Ascorbate → 2 Fe^2+^ + Dehydroascorbate (4)

2 Fe^2+^ + 2 H_2_O_2_ → 2 Fe^3+^ + 2 OH**·** + 2 OH^−^ (5)

The estimated half-life of the same ROS is very short (e.g., for OH is 10^−9^ s), which means that low weight synthetic antioxidants (e.g., vitamins C, E) are unable to specifically scavenge such ROS in vivo due to their extreme reactivity [118,119]. In contrast, enzymatic antioxidants have the ability to accelerate chemical reactions. This ability to eliminate ROS is, therefore, much higher than that of low molecular weight antioxidants ingested through the diet. Therefore, activation of enzymatic antioxidant defenses in vivo is more relevant than radical scavenging by exogenous antioxidants consumed through the diet [120].

Although there are no official guidelines regarding the necessary quantity of antioxidants [121], it is recommended to primarily follow a diverse and balanced diet. Daily consumption of at least 400 g of fruits and vegetables is recommended by the World Health Organization to gain adequate protective compounds from the normal diet to scavenge ROS.

Since preventing the formation of ROS is considered to be more effective in reducing oxidative stress than the suppression of already created free radicals with antioxidants, metal chelating antioxidants (e.g., transferrin, albumin, ceruloplasmin) could be used to prevent radical formation by inhibiting the Fenton reaction catalyzed by transition metal ions. In addition, various flavonoids act as chelators of transition metal ions involved in Fenton chemistry [122,123,124].

In any case, the justification and appropriateness of supplemental intake of antioxidants or topical application of antioxidants should be confirmed by the results of human studies that would establish the most effective antioxidants, the most appropriate concentration, combination and timing of intake of antioxidants in patients treated with non-removable orthodontic appliances. Antioxidant therapy during dental treatment proved to be effective in the reduction of oxidative stress and cytotoxicity during tooth whitening and in restorative dentistry (reviewed in [125]).

Another approach that may be effective in preventing oxidative stress in the oral cavity is the direct application of antioxidants to metal parts of orthodontic appliances, which could intercept free radicals directly in the vicinity of their formation before they can damage surrounding tissues. On the other hand, the application of antioxidants to the metal parts of orthodontic appliances may lead to the damage of the protective layer, corrosion, and increased leaching of metal ions. Further research into these domains is, therefore, to be undertaken.

## 8. Conclusions

The broad topic was considered from many different perspectives, as the narrative review was divided into various sections. To summarize: The results of in vitro and in vivo studies that examined the release of metal ions show differences in the amount of metal ions released due to different study designs. Metal ions released from corroded orthodontic appliances could be exposed to redox cycling reactions and cause oxidative stress. Therefore, the safety of such appliances should be investigated, the risk assessed, and the justification and appropriateness of additional antioxidant intake confirmed by the results of future human studies to demonstrate the efficacy of antioxidant therapy in reducing oxidative stress during dental treatment. The consumer growing awareness, interest and knowledge impels the manufacturing industry to confront the challenges of choosing adequate orthodontic appliance materials to minimize their impact on health.

Although Žukowski et al. [126] reported some beneficial effects of generating ROS in the oral cavity during dental treatment (e.g., stimulation of immune response and wound healing, cytotoxic effect on pathogenic bacteria), the metal ions released into the oral cavity from fixed orthodontic appliances due to corrosion and biodegradation pose a health risk to patients. This risk is, however, a minor one, as only very high metal concentrations induce cytotoxicity and oxidative stress, which was found in the studies on cell cultures. On the other hand, several studies reported that orthodontic treatment can induce transient imbalance in the ratio between oxidants and antioxidants in saliva as well as at the systemic level of clinically healthy subjects.

Nevertheless, the increased ROS levels can occur at a local level in the oral cavity, which could pose a problem to patients with lower efficiency of endogenous antioxidant defense systems, metal ions hypersensitive individuals, or patients with decreased salivary antioxidant power [127]. This topic should be further investigated, since there is currently only scarce scientific research on the effect of orthodontic materials on the formation of oxidative stress.
